# Insights into host dependency from a chemically defined medium for the human vaginal bacterium *Lactobacillus crispatus*

**DOI:** 10.1007/s00203-025-04406-z

**Published:** 2025-08-06

**Authors:** P. Achterberg, N. J. K. van Geenen, R. Y. Hertzberger, D. Molenaar, M. C. M. van Loosdrecht, R. Kort

**Affiliations:** 1 Amsterdam Institute for Life and Environment (A-LIFE), Vrije Universiteit Amsterdam, De Boelelaan 1108, 1081 HZ Amsterdam, The Netherlands; 2 Department of Biotechnology, Delft University of Technology, van der Maasweg 9, 2629 HZ Delft, The Netherlands; 3 ARTIS-Micropia, Plantage Kerklaan 38-40, 1018 CZ Amsterdam, The Netherlands

**Keywords:** *Lactobacillus crispatus*, *Lactobacillaceae*, Vaginal microbiome, Nutrient requirements, Chemically defined medium

## Abstract

A *Lactobacillus*-rich vaginal microbiome is associated with a reduced risk for sexually transmitted diseases and adverse reproductive health outcomes, with *Lactobacillus crispatus* identified as particularly beneficial. This study investigated the nutritional requirements of two vaginal isolates, *L. crispatus* RL09 and RL10, and presents a chemically defined medium (CDM) that supports their growth. This study experimentally validated that *L. crispatus* relies on exogenous fatty acids, essential B vitamins, including riboflavin (B2), niacin (B3) and pantothenate (B5), and all eighteen amino acids for sustained growth. On one hand, this body of work adds to the physiological understanding of *Lactobacilliaceae* and lays the groundwork for future quantitative studies on *L. crispatus*. On the other hand, we have shown that *L. crispatus* exhibits a high metabolic dependency on its environment. These dependencies underscore the potential sensitivity of *L. crispatus* to nutrient variations, which may influence its ability to dominate and maintain a healthy vaginal ecosystem.

**Supplementary Information:**

The online version contains supplementary material available at 10.1007/s00203-025-04406-z.

## Introduction

The eubiotic vaginal microbiome is predominantly composed of one of four lactobacilli: *L. crispatus, L. gasseri, L. jensenii* or *L. iners*, all of which are part of the obligate homofermentative *L. delbrueckii* group, currently known as the only *Lactobacillus* genus (Duar et al. 2017; Zheng et al. 2020). A lactobacilli-*rich* vaginal microbiome is associated with a reduced risk for sexually transmitted diseases and adverse reproductive health outcomes, with *L. crispatus* identified as particularly beneficial (France et al. 2022).

*Lactobacillaceae* are extensively studied for their economic value, especially in food and feed fermentations (Duar et al. 2017). Previously, chemically defined media (CDM) have been developed for *Lactobacillaceae* and used to study their metabolic capacity. These include CDMs for *Lactobacillus acidophilus* (Lv et al. 2017; Meng et al. 2021*), Lactobacillus helveticus* (Morishita et al. 1981; Hébert et al. 2000*), Lactobacillus delbrueckii* subsp*. bulgaricus* (Chervaux et al. 2000*), Lactobacillus delbrueckii* subsp. *lactis* (Hébert et al. 2004a), *Limosolactobacillus reuteri* (Santos 2008; Kwoji et al. 2022*), Lacticaseibacillus casei* and *Lactiplantibacillus plantarum* subsp. *plantarum* (Morishita et al. 1981).

These and other studies show that *Lactobacilliaceae*, particularly the often host-adapted lactobacilli, are auxotrophic for many key nutrients, reflected in their smaller genome sizes (Makarova and Slesarev 2006). In *Lactobacillaceae*, the genome sizes of host-adapted organisms are significantly smaller than their more ‘nomadic’ counterparts (Duar et al. 2017; Morishita et al. 1981). Adaptation to nutrient-rich host environments coincided with the loss of metabolic potential, transforming some of these organisms into obligate symbionts with specific nutrient requirements. Vaginal-isolated *L. crispatus* is among the more degenerative *Lactobacilliaceae* with a genome size of around 2.2 Mb (Duar et al. 2017; Pan et al. 2020a) compared to the 3.45 Mb of the nomadic *L. plantarum* subsp. *plantarum* (Zheng et al. 2020). It should be noted that several other species from the *Lactobacillus* genus, including the other key vaginal species have smaller genome sizes than *L. crispatus* (Pan et al. 2020a; Lebeer et al. 2023*).*

Despite its crucial role in the vaginal ecosystem, the detailed nutrient requirements and metabolic capacity of *L. crispatus* are not fully understood. Common media for cultivating *L. crispatus* are Man, Rogosa and Sharpe broth (MRS) and New York City III broth (NYCIII) (Navarro et al. 2023). These complex media contain undefined compounds such as beef extract and horse serum to meet the fastidious nutrient requirements. While they sustain the growth of *L. crispatus* they limit or increase the complexity of analytical methods to study its physiology***.*** A completely chemically defined medium that supports the growth of *L. crispatus* would aid future physiological studies. At the same time, an improved understanding of the nutrient requirements and physiological needs of *L. crispatus* could potentially be leveraged for novel therapeutic purposes. Currently, only limited interventions, to shift a dysbiotic vaginal microbiome towards a *Lactobacillus (crispatus)* dominant one, are available on the market, e.g. LACTIN-V following an antibiotic treatment (Hemmerling et al. 2024).

This study aims to elucidate the nutritional requirements of *L. crispatus* and to develop a (minimal) chemically defined medium (CDM) that supports biomass growth and acidification. This will be achieved through single- or multiple-omission growth experiments with the previously isolated human vaginal bacteria *L. crispatus* RL09 and RL10 (van der Veer et al. 2019). Additionally, the growth characteristics of the CDM and a redefined CDM will be evaluated to assess the medium’s applicability.

## Materials and methods

### Bacterial strains and (initial) growth conditions

The vaginal isolated *Lactobacillus crispatus* RL09 and RL10 strains used throughout this study were obtained in previous work described in papers by van der Veer et al. (2019), Dols et al. (2016). They were isolated from a *L. crispatus* dominant and a *L. iners* dominant vaginal microbiome, respectively.

All bacterial growth experiments were performed anaerobically at 37 °C. Anaerobic conditions were maintained in anaerobic jars (Oxoid modified 3, HP0011a or an airtight metal jar) with anaerobic gas-generating sachets (AnaeroGen 2.5 or 3.5L; ThermoScientific, Waltham, MA, USA) added before sealing. Handling of the bacterial cultures, including inoculation and transfers, was performed under sterile conditions using standard aseptic techniques under ambient atmospheric conditions. Immediately after handling, cultures were transferred to anaerobic jars to minimise oxygen exposure. Throughout all experiments and each procedural step, negative controls were included and inspected for microbial contamination.

Bacterial cultures were propagated from − 70 °C NYCIII or MRS glycerol stocks (~ 20% w/w) on NYCIII agar (1.5%) plates for at least 48 h. Pre-cultures of 2 mL NYCIII were inoculated from the plate and incubated for 22-26 h in screw-cabbed tubes with loose lids to allow gas transfer between the tubes and the jar. The inoculation plates and pre-cultures were always freshly prepared prior to each growth experiment.

### NYCIII medium (liquid and plates)

The NYCIII medium was adjusted from the ATCC medium (1685). For 1L of medium, the following ingredients were added: 3.8 g Oxoid™ yeast extract (Oxoid Ltd., Basingstoke, United Kingdom; LP0021B), 5.5 g (d-) glucose monohydrate (Millipore^®^, Merck, Darmstadt, Germany), 2.4 g HEPES (Sigma Aldrich, Burlington, MA, USA; > 99.5%), 15 g Bacto™ proteose peptone No.3 (BD, Sparks, MD, USA), 5.0 g sodium chloride (> 99%, Sigma Aldrich), 10% v/v horse serum (filter sterilised and heat-inactivated, Gibco, Grand Island, NY, USA), demineralised water. In the case of agar plates, 15 g/L of agar (for bacteriology, VWR) was added. The liquid was sterilised by autoclavation at 121 °C for 15 min. The horse serum was added under sterile conditions when the media cooled down sufficiently (approx. 37◦C to prevent denaturation of the proteins). Plates and liquid media were stored at 4◦C until further usage.

### Cultivation media and experimental set-up for the omission experiments

The complete chemically defined medium (CDM, Table 1) was originally developed for *Limosilactobacillus reuteri* by Filipe Branco dos Santos (2008). Stock solutions of different CDM parts were made and stored in the fridge or freezer until further usage. A detailed protocol for preparing the various stock solutions is available upon request. The basal solution, containing the glucose, was filter sterilised (0.22 µm PES membrane) before storage. All used chemicals were at least reagent grade (≥ 95%). When the various CDM media were prepared for the growth experiments, the solutions from the freezer were thawed on the bench and added together to obtain the concentrations as shown in Table 1. Directly after preparing the final CDM the solutions were filter sterilised with a 50 ml syringe and 0.22 µm PES membrane filter and used the same day or stored in the fridge to be used within a week.

**Table 1:** Composition of the complete chemically defined medium (CDM) and the redefined medium (redefCDM)

Compound	Concentration (g/L)	
	CDM	redefCDM
Dipotassium hydrogen phosphate	1	1
Glucose monohydrate	11	11
Iron(II)sulfate heptahydrate	0.02	
l-ascorbic acid	0.5	
Magnesium sulfate heptahydrate	0.2	0.2
Manganese(II)sulfate tetrahydrate	0.05	0.05
Monopotassium hydrogen phosphate	5	5
Sodiumacetate	1	1
Triammonium citrate	0.8	0.8
Tween80	1*	1*
l-Alanine	0.24	0.24
l-Arginine	0.125	0.125
l-Aspartic acid	0.42	0.42
Cysteine-HCl	0.13	0.13
Glutamic acid	0.5	0.5
Glycine	0.175	0.175
l-Histidine	0.15	0.15
l-Isoleucine	0.21	0.21
l-Leucine	0.475	0.475
l-Lysine	0.44	0.44
l-Methionine	0.125	0.125
l-Phenylalanine	0.275	0.275
l-Proline	0.675	0.675
l-Serine	0.34	0.34
l-Threonine	0.225	0.225
l-Tryptophane	0.05	0.05
l-Tyrosine	0.25	0.25
l-Valine	0.325	0.325
Alpha-lipoic acid	0.0025	
Ca-(D)-pantothenate	0.001	0.001
D-Biotin	0.0025	
Folic acid	0.002	0.002
Inosine	0.005	
Nicotinic acid	0.001	0.001
Orotic acid	0.005	0.005
*p*-Aminobenzoëic acid	0.005	
Pyridoxamine HCl	0.005	0.005
Pyridoxine–HCl	0.002	
Riboflavin	0.001	0.001
Thiamin-HCl	0.001	
Thymidine	0.005	
Adenine	0.01	0.01
Guanine	0.01	
Uracil	0.01	
Xanthine	0.01	0.01

* ml/L

The single- or multiple omission experiments were performed in sterile Cellstar^®^ 48-well plates (Greiner Bio-one, Kremsmünster, Austria). For the first propagation, 980 µl of media and 20 µl of pre-culture or sterilised milliQ for the controls was added to the wells. After 24 h of incubation, the second propagation was prepared by adding 980 µl of media and 20 µl cell broth of the same condition as the first propagation to a new 48-well plate. At the same time 490 µl cell broth of the first propagation was transferred for analysis, and the remaining 490 µl was incubated another 24 h to create a 48 h time point. In case of a third propagation, this was repeated with inoculate from the second propagation. The third propagation was only incubated for 24 h. For each experimental condition, a minimum of two and most often three biological replicates were performed, each with two technical replicates, resulting in a total of 4–6 data points per condition.

### Analytical methods

After 24 or 48 h of bacterial growth, the pH was measured with the HI 2210 pH meter (Hanna Instruments, Nieuwegein, Netherlands) at roomtemperature (21–22 °C). The optical density at 600 nm (OD600nm) was measured with a microplate reader FluoStar Omega (BMG Labtech, Ortenberg, Germany), Infinite® 200PRO (Tecan Life Sciences, Männedorf, Switzerland) or BioTek Synergy HTX multimode reader (Agilent Technologies, Santa Clara, CA, USA). The same instrument was used for the duration of one growth experiment. The lid of the 48-well plate was kept on. Dilutions were made with phosphate-buffered saline (PBS, Sigma-Aldrich). Next, 1–2 ml of the cell broth was spun down for five minutes at 10,000 rpm at 10 °C (Heraeus Fresco 17 Centrifuge, ThermoScientific) and the supernatant was stored at − 20 °C.

High-performance liquid chromatography (HPLC) was performed with the obtained supernatant, after filtering with a 0.22 µm PES filter, to determine glucose, succinate, lactate, formate, glycerol, acetate, propionate, butyrate, and valerate. The Vanquish HPLC system (ThermoScientific) was equipped with an Aminex HPX-87 column (BioRad, Hercules, CA, USA) maintained at 50◦C and coupled to an ultraviolet (UV, 210 nm) and refraction index (RI) detector. The eluent consisted of 1.5 mM phosphoric acid and had a flow rate of 0.75 ml/min. Integration by the software, Chromeleon 7 (ThermoScientific), was checked manually and if needed adjusted. Validation of the compounds was done based on the RI spectrum only, as some compounds in the medium overlapped on the UV spectrum. Calibration was done on the following compounds with glucose-D-monohydrate (‘Baker Analysed’, VWR), ethanol (99.8%, Honeywell), Volatile fatty acid mix (Sigma-Aldrich), succinic acid (99.0%, SigmaUltra), l-Lactic acid (80%, Sigma-Aldrich).

### Data analysis

Raw data from the pH and OD600nm measurements were collected in Microsoft Excel. Data analysis and visualisations were performed using custom scripts written in R (version 4.3.0) using Rstudio (version “Mountain Hydrangea” Release 583b465e, 2023-06-05) and the loaded R libraries: tidyverse 2.0.0, readxl 1.4.3. For each growth experiment, a minimum of two biological replicates was performed. The increase in OD600nm (ΔOD600nm) for a sample was determined by correcting the measured OD600nm with the used dilution and subtracting the measurement of the control (cell-free cCDM), which was averaged in case of technical duplicates.

## Results

To determine the nutritional requirements of the human vaginal bacterium *Lactobacillus crispatus* RL09 and RL10 single- or multiple-omission experiments were performed with most components from the complete chemically defined medium (CDM; Table 1). The optical density (OD600nm) was used as a proxy for bacterial growth, and acidification measured by pH was used to validate the observed results.

### Essential major components to sustain growth

**Fig. 1:**
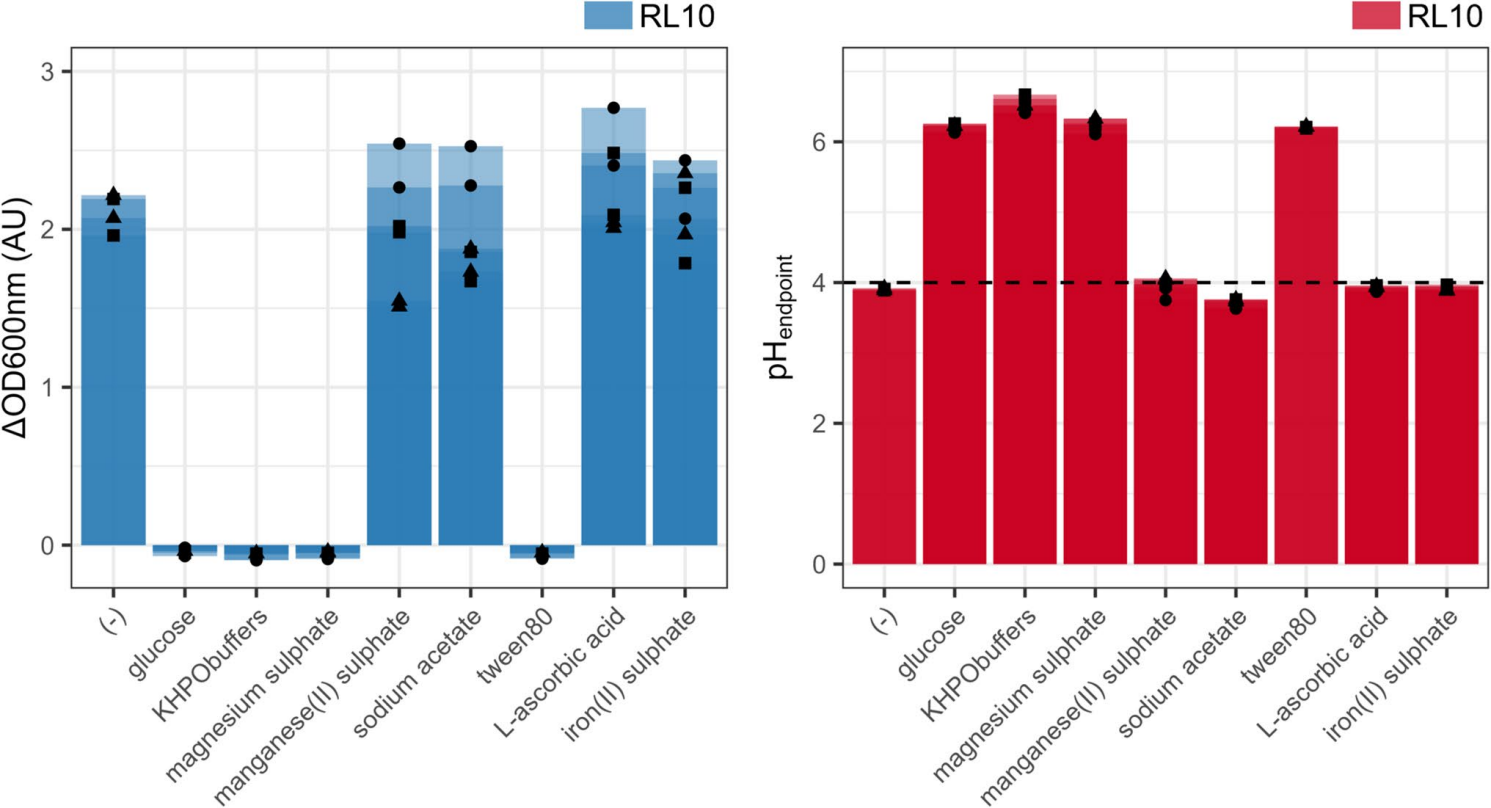
The effect of single and multiple omission of basal components of the chemically defined medium on the growth of L. crispatus RL10. The *X* axis depicts the omitted nutrient, with (−) being the complete CDM. Left: the *Y* axis depicts the increase in optical density at 600 nm (ΔOD600 nm). Right: the *Y* axis represents the measured pH at the end of the batch (pH_endpoint_). Three biological replications are shown (●, ▲ and ■), including their technical replicates. Solely the second propagation at 24 h is shown, results of the first and third are available in the supplementary data (Sup. Figure 1)

Glucose, Tween80, magnesium sulphate (MgSO_4_) and potassium phosphate (KH_2_PO_4_ and K_2_HPO_4_) were observed to be essential for growth and acidification (Fig. 1, Supplementary Figure S1). Eliminating iron(II)sulphate (FeSO_4_) did not impact growth or acidification. Omission of sodium acetate and manganese(II)sulphate (MnSO_4_) decreased biomass growth slightly, the difference compared to the complete CDM was more profound at 48 h.

### *Lactobacillus crispatus* RL10 and RL09 need all amino acids for sustained growth

**Fig. 2:**
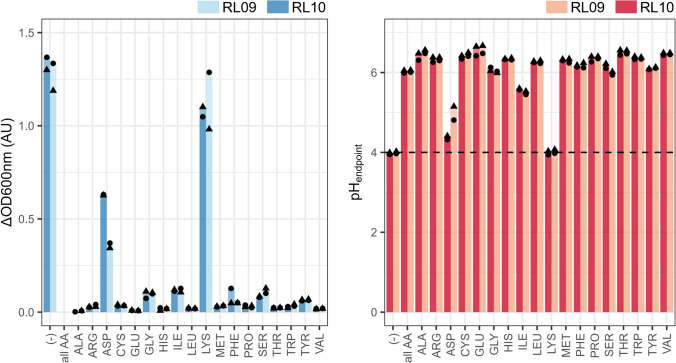
The effect of the single or complete omission of amino acids of the chemically defined medium on the biomass growth and acidification of *L. crispatus* RL09 and RL10. The X-axis depicts the omitted nutrient, with (−) being the complete CDM. Left: the *Y* axis depicts the increase in optical density at 600 nm (ΔOD600 nm). Right: the Y-axis represents the measured pH at the end of the batch (pH_endpoint_). Two biological replications (● and▲) are shown. Solely the second propagation at 24 h is shown, results of the first and third are available in the supplementary data (Sup. Figure 2)

**Fig. 3:**
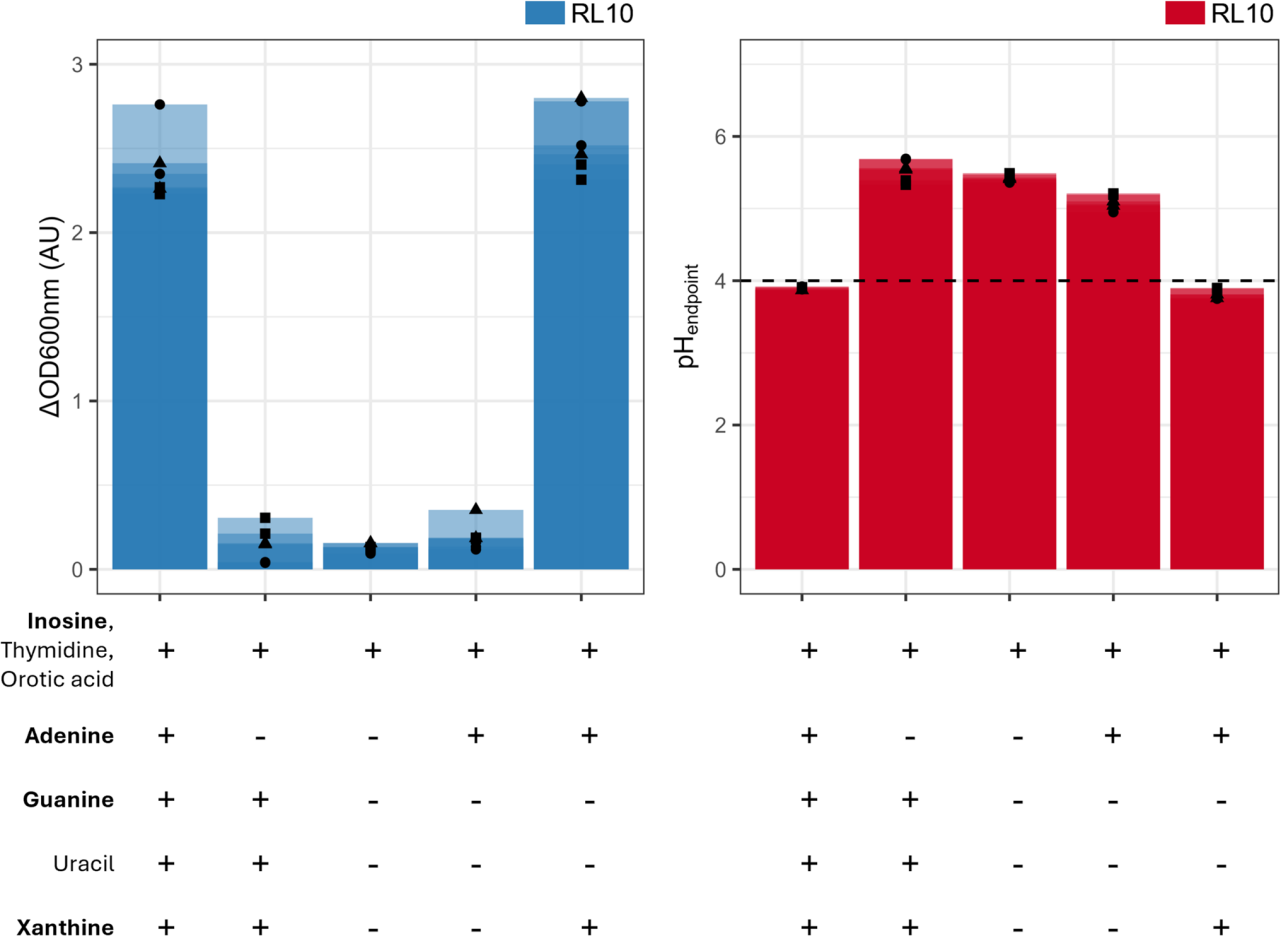
The effect of the single or complete omission of nucleotides of the chemically defined medium on the biomass growth and acidification of *L. crispatus* RL10. The X-axis depicts the omitted nutrient, with (−) being the complete CDM. In bold the purines and in normal font the pyrimidines. Left: the *Y* axis depicts the increase in optical density at 600 nm (ΔOD600 nm). Right: the *Y* axis represents the measured pH at the end of the batch (pH_endpoint_). Two biological replications (●, ▲ and ■) are shown including their technical replicates. Solely the second propagation at 24 h is shown, results of the first and third are available in the supplementary data (Sup. Figure 3)

**Fig. 4:**
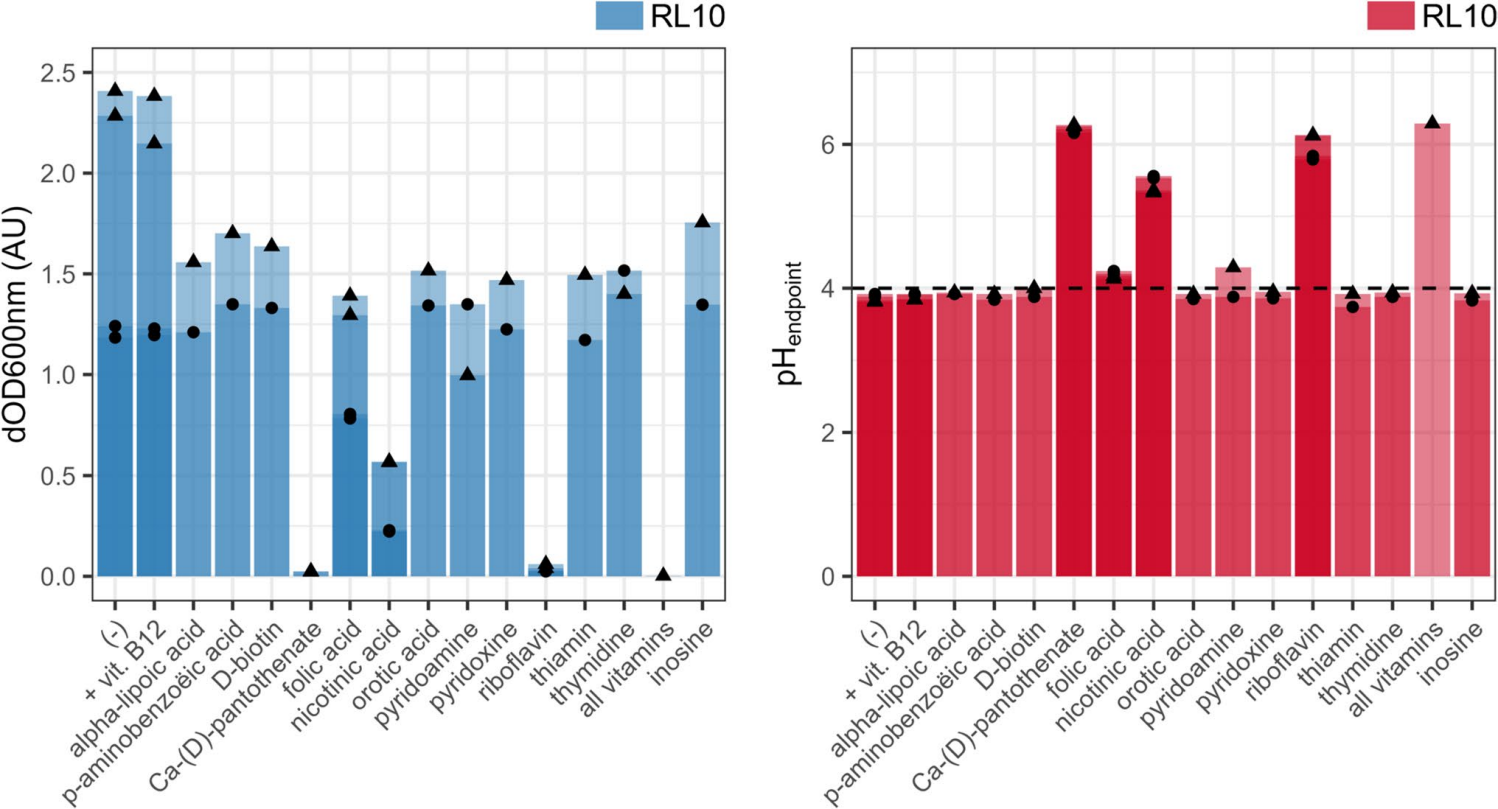
The effect of the single or complete omission or addition (vit. B12) of vitamins/nucleotides of the chemically defined medium on the biomass growth and acidification of *L. crispatus* RL10. The *X* axis depicts the omitted nutrient, with (-) being the complete CDM or the addition to the cCDM for vit. B12. Left: the *Y* axis depicts the increase in optical density at 600 nm (ΔOD600 nm). Right: the Y-axis represents the measured pH at the end of the batch (pH_endpoint_). Two biological replications (● and▲) are shown, including their technical replicates. Solely the second propagation at 24 h is shown, results of the first and third are available in the supplementary data (Sup. Figure 4)

The complete CDM comprises of 18 amino acids; glutamine (GLN) and asparagine (ASN) are excluded from this media. Their acidic counterparts, respectively glutamic acid (GLU) and aspartic acid (ASP) are included. Single-omission of the amino acids indicated that all are essential to support sustained growth for both *L. crispatus* RL09 and RL10 (Fig. 2; Sup. Fig. 2). Removal of aspartic acid (ASP) or lysine (LYS) yielded a less severe growth reduction in both strains and are therefore considered stimulatory. Limited growth was observed with the omission of some amino acids, namely, glycine (GLY), serine (SER), isoleucine (ILE), Tyrosine (Tyr) (Figs. 3, 4).

### Nucleotides and their precursors impact growth

**Fig. 5:**
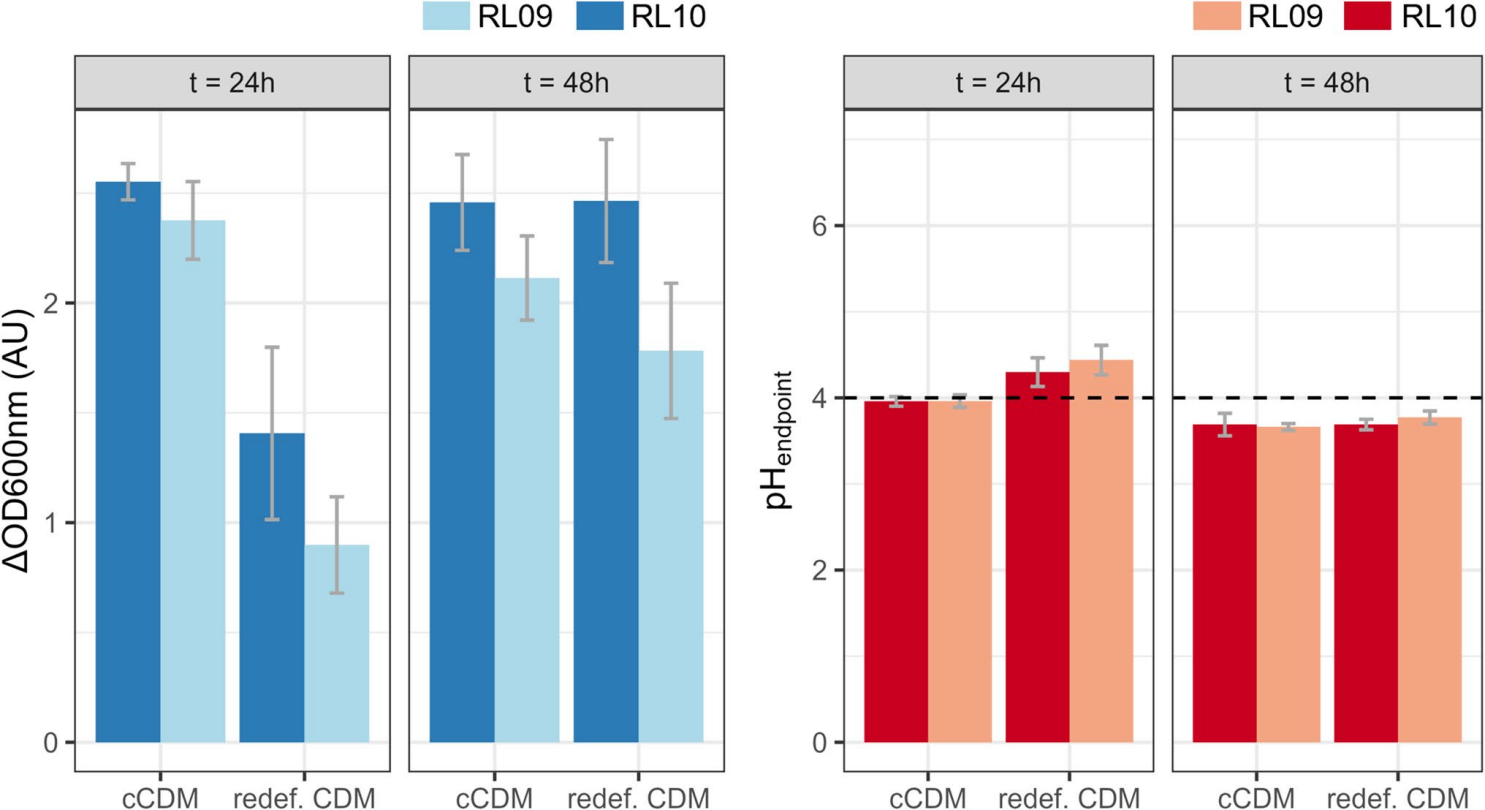
Complete CDM v.s. redefined CDM at the 2nd propagation at 24 h and 48 h of growth with *L. crispatus* RL09 and RL10. They were grown anaerobically at 37 °C on complete CDM and redefined CDM (*X* axis), and the optical density at 600 nm was measured at 24 and 48 h (*Y* axis). The error bars depict the standard deviation of three biological replicates, with each two technical replicates

The CDM contained four purines; adenine, guanine, inosine, xanthine and three pyrimidines; orotic acid, thymidine and uracil for nucleotide bases and precursors. The single omission of thymidine, orotic acid and inosine showed no effect(Fig. 5). Thymidine with orotic acid or solely uracil can fulfil the need for pyrimidines. For the purines, supplementation of inosine alone is not sufficient as this impacts growth immensely (Fig. 4). Addition of adenine and xanthine, next to inosine restored the growth profile of the complete CDM. Single omission of adenine did support growth but altered the growth rate significantly, this is also the case when guanine and xanthine are omitted but to a lower extend (Supl. Fig. 4).

### Riboflavin, nicotinic acid and Ca-(d) pantothenate, are essential for proliferation

Of the eleven vitamins present in the CDM, three proved to be essential for *L. crispatus* RL10, namely riboflavin (vit. B2), nicotinic acid (vit. B3) and Ca-(D)-pantothenate (vit. B5) (Fig. 1 and 4; Sup. Fig. 1 and 4). The omission of thiamine, pyridoxamine, folic acid and biotin (vit. B8), pyridoxine, alpha lipoic acid, and p-aminobenzoic acid (PABA) resulted in a slight growth reduction. l-ascorbic acid (vit. C) could be omitted from the medium without impacting the growth. Vitamin B12 was not present in the original CDM and was not needed for growth, the addition of vitamin B12 did not alter the measured growth pattern.

### Elimination of non-essential compounds reduces growth and acidification rate

Based on the nutritional requirements observed from the single- and multiple-omission experiments a redefined chemically defined medium (redefCDM) was developed (see Table 1). Growth of *L. crispatus* RL09 and RL10 on the complete CDM and the redefined CDM was studied (Fig. 2).

Elimination of L-ascorbic acid (vit. C), iron(II)sulphate heptahydrate, uracil, guanine, thymidine, inosine, D-biotin, alpha-lipoic acid, pyridoxine–HCl, thiamine-HCl, and p-aminobenzoic acid results in a reduction in growth rate for the *L. crispatus* RL strains. After 48 h, the same optical density and acidification are obtained as on the complete CDM. High-performance liquid chromatography analysis showed that lactic acid (LA) levels reached approximately 100 mM, with a yield (Y_S/P_) of around 2 mmol LA/mmol glucose converted.

## Discussion

This study explored the nutritional requirements and growth behaviour of the vaginal bacteria *L. crispatus* RL10 and RL09, presenting both a chemically defined medium (CDM) and redefined medium (refCDM) that reliably support their sustained growth. As the introduction highlights, several CDMs have been developed for various species within the *Lactobacilliaceae* family, and numerous studies have examined nutrient dependencies across this group. The observed auxotrophies in *L. crispatus* are similar, if not identical, to those reported for related *Lactobacillus* species (Salvetti et al. 2013, 2018). Insufficient levels of essential nutrients may compromise the capacity of *Lactobacillus crispatus* to sustain its dominance within the vaginal microbiome, creating an ecological niche for pathogenic bacteria to proliferate. This discussion will focus on critical nutrient dependencies most likely to influence the vaginal ecosystem, highlighting their potential for therapeutic application.

### The role of B-vitamins in the vaginal ecosystem

Many lactic acid bacteria (including *Lactobacillaceae*) are auxotrophic for certain B-vitamins (Magnúsdóttir et al. 2015; Le Blanc et al. 2011). Our results align with these observations, validating that riboflavin (vit. B2), nicotinic acid (vit. B3) and Ca-(d)-pantothenate (vit. B5) are also required for the growth of *L. crispatus*. It is potentially also auxotrophic for folate (vit. B9) and pyridoxin. However, as folic acid and pABA were not omitted simultaneously, it cannot be concluded that this compound is required. The same holds for pyridoxine and pyridoxamine.

Deficiencies in some B-vitamins (e.g. riboflavin, folate) in humans are prevalent even in seemingly healthy cohorts (Sivaprasad et al. 2019). Only limited literature is available that looks at the impact of B-vitamins on the vaginal microbiome. Tuddenham et al. (2019) looked at the association between several dietary micronutrients, including some B-vitamins and the prevalence of bacterial vaginosis (BV) by 16sRNA fingerprinting. They did not find a significant correlation between BV and folate, niacin (vit. B3), or B6. Dunlop et al. (2011) indicated that folate deficiency is related to maternal BV (Dunlop et al. 2011), and similarly a study by Cui et al. (2023) reported that increased serum and red blood cell (RBC) folate were associated with a decreased risk of bacterial vaginosis (BV) (Cui et al. 2023). The latter explains this by folate’s enhancing impact on the immune system and response. However, folate might also directly influence the proliferation of lactobacilli in the vaginal ecosystem, although this has not been studied to date.

A recent literature study underscored the importance of riboflavin, one of the identified essential vitamins, in women’s health (Dricot et al. 2024). Riboflavin (vitamin B2) is one of the identified essential vitamins, yet its role in the vaginal microbiome has not been extensively studied. Interestingly, a study by Lebeer et al. (2023) found a positive correlation between *L. crispatus* dominance and the presence of *Limosolactobacillus* spp. and they found that an isolated strain from a vaginal swap of this taxon produced significant amounts of riboflavin (Spacova et al. 2022). They recently started an in vivo study, VIAB_2_L (Isala 2024), which will investigate the effect of riboflavin supplementation on the vaginal microbiome. Ca-D-pantothenate (vit. B5), another essential vitamin for *L. crispatus* growth has not been included in studies regarding the vaginal microbiome. Deficiencies in this compound are rare, and only occur in severe malnutrition; hence it is unlikely that this compound is affecting the vaginal microbiome in seemingly healthy humans (Zempleni 2007). In summary, the role of certain B-vitamins, e.g. folate and riboflavin, in supporting *L. crispatus* proliferation and in shaping the vaginal microbiome remains an intriguing area of research that warrants further investigation.

### Oleic acid in Tween80 fulfils the need for an exogenous source of (unsaturated) fatty acid

This study experimentally validated that the nonionic surfactant Tween80 is essential for sustained growth and acidification of *L. crispatus* RL10. Tween80 is a polyethylene sorbitol ester containing approximately twenty ethylene oxide units, one sorbitol, and one oleic acid (C_18:1 cis-9_) as the primary fatty acid (Sigma-Aldrich TWEEN^®^ 2025). Although the precise component of Tween80 responsible for the growth dependence was not confirmed here, prior studies with *Lactobacillus* spp. identified oleic acid as the essential nutrient, which serves as an exogenous source of unsaturated fatty acid for the bacteria (Partanen et al. 2001; Thompson et al. 2024; Zhu et al. 2024). This dependency is consistent with genome analyses that identified that *L. crispatus* and othervaginal lactobacilli, such as * Lactobacillus johnsonii, L. gasseri*, lack an intact FASII fatty acid biosynthesis pathway (Makarova and Slesarev., A. 2006; Zhu et al. 2024). Consequently, these bacteria rely on exogenous fatty acids to support growth, a need met by oleic acid in Tween80. It is hypothesised that within the NYCIII medium, the horse serum fulfils the need for an exogenous source of fatty acid for *L. crispatus*.

The absence of genes encoding enzymes in the FASII pathway is likely a result of host adaptation. Oleic acid is widespread and abundantly present in mammalians including human mucosal surfaces, hence it is most likely available in the vaginal environment (Khoury et al. 2021; Xie et al. 2024; Calder 2015). Variations in the fatty acid composition of the bacterial membrane influence its biophysical properties, such as fluidity, permeability, and structural stability, which are critical components of stress resistance, including survival in acidic environments. For example, it was observed that *L. plantarum* incorporates more of the unsaturated oleic acid in its membrane upon exposure to an acidic environment (Xia et al. 2023). Given that *L. crispatus* (and other vaginal lactobacilli) cannot synthesise endogenous fatty acids, their survival and physiological state are highly dependent on the availability and type of exogenous fatty acids provided in the vaginal ecosystem. Long-chain fatty acids (LCFAs) may play a key role in the shaping of the vaginal microbiome. A recent study, by Zhu et al., provided evidence that oleic acid and other similar LCFAs inhibited *L. iners* while promoting *L. crispatus*, supporting idea that these compounds potentially serve as selective modulators in the vaginal microbiome (Zhu et al. 2024).

### *Lactobacillus crispatus* exhibits a greater degree of amino acid auxotrophy within the *Lactobacillaceae* family

Numerous members of the *Lactobacillaceae* family cannot synthesise certain amino acids de novo (Table 2)*.* However, the requirements vary widely and tend to reflect adaptation to host-associated environments, often rich in proteins (Duar et al. 2017; Ramoneda et al. 2023). For instance, *L. plantarum* WCFS1 requires nine amino-acids (Wegkamp et al. 2010) and *L. helveticus* ATCC 15009 fourteen (Morishita et al. 1981). Notably, intraspecies variability exists as highlighted for both these and other organisms (Hébert et al. 2000; Wegkamp et al. 2010). This study revealed that *L. crispatus* RL09 and RL10 depend on all eighteen amino acids in the CDM for sustained growth. However, aspartic acid and lysine are not strict auxotrophies, as their omission still allowed growth, but with reduced biomass.

**Table 2:**
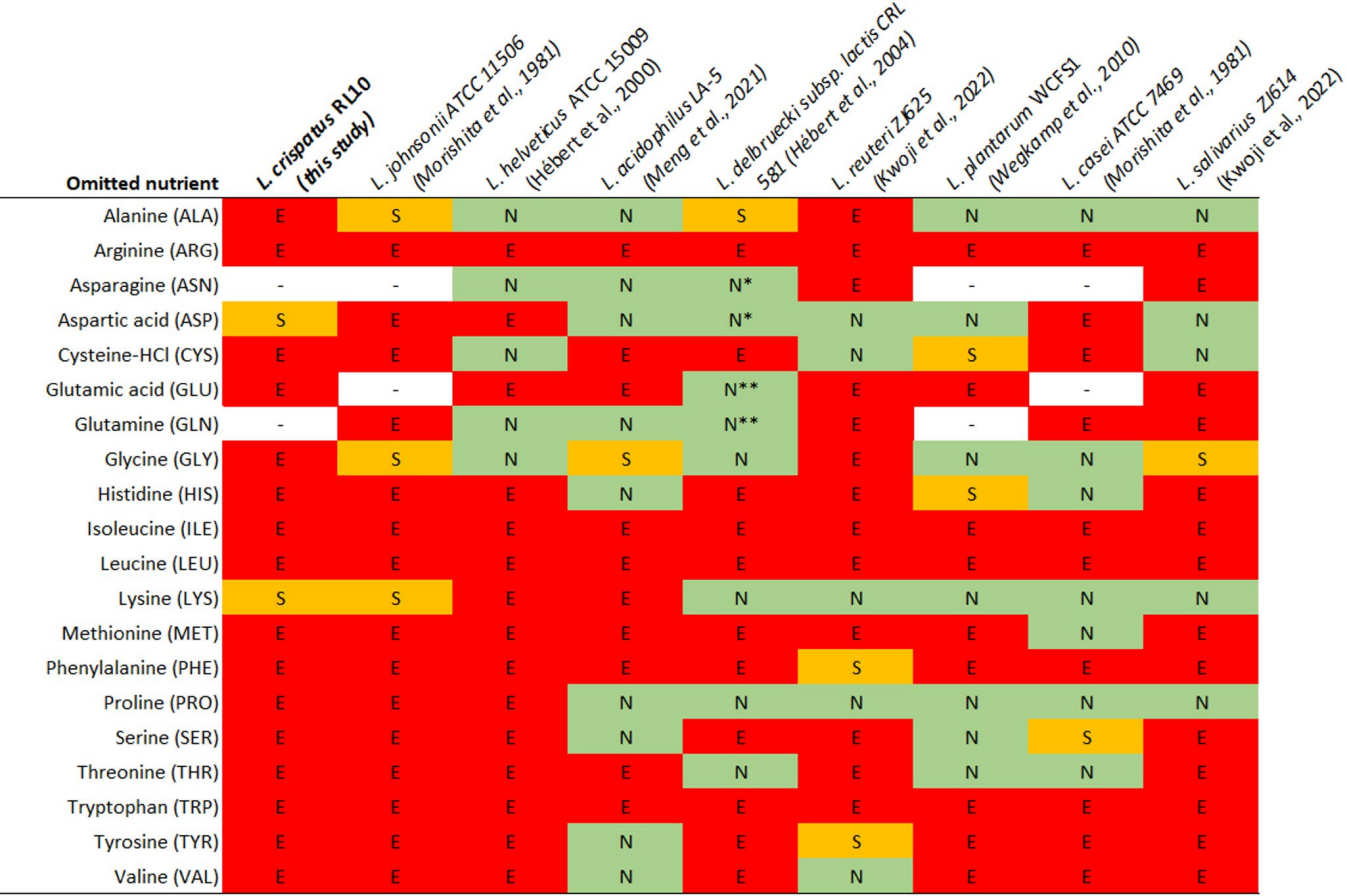
Amino acid requirement for selected *Lactobacillaceae*

Green (N) indicates the compound is non-essential, orange (S) that the amino acid is stimulatory and red (E) essential, when omission results in no or extremely poor growth. ‘−‘ Indicates that this compound wasnot evaluated. * When ASN and ASP are omitted together, the requirement is stimulatory. ** When GLUand GLN are omitted simultaneously, no growth is observed

The alanine and glycine dependencies in *L. crispatus* RL09 and RL10 are rarely observed within the *Lactobacillaceae* family, however, some instances have been documented in the literature. For example, *L. reuteri* ZJ614 has been reported to require alanine and glycine, although it does not exhibit the same dependencies for other amino acids (Kwoji et al. 2022). Similarly, *L. johnsonii* (found in the mammalian digestive tracts) was shown to be auxotrophic for alanine in both wet-lab and genomic analysis (Kaaij et al. 2004). The complete amino acid dependencies of *L. crispatus* RL09 and RL10 were found to match with only one other study, namely one by Morishita et al. (1981) for *L. johnsonii* ATCC 11506 (in the article reported as *L. acidophilus* ATCC 11506) (Morishita et al. 1981). Hence, the results suggest that *L. crispatus* has more extensive amino acid requirements than closely related species, such as *L. helveticus* and *L. acidophilus*, most likely reflecting its adaptation to the nutrient availability in the vaginal environment. However, due to the limited studies examining amino acid dependencies in related (vaginal) species from the obligate homofermentative *Lactobacillus* genus (e.g. *L. gasseri, L. iners*, *L. jensenii*, *L. amylovorus*), this view might be biased and incomplete. Future studies employing wet-lab experiments and genomic analysis could clarify whether these extensive auxotrophies are unique to *L. crispatus* or shared with other (vaginal) *Lactobacillus* species. All amino acids, except for asparagine, were detected in cervicovaginal lavage fluid in a study by Srinivasan et al. (2015). The same study reported that women with bacterial vaginosis (BV) exhibited lower concentrations of amino acids compared to healthy individuals. While this difference may reflect the higher bacterial load typically associated with BV (Armstrong et al. 2025), it could also indicate a shift available amino acids in the vaginal environment. Additionally, a study by Bloom et al. (2022) highlighted that *L. iners,* compared to *L. crispatus*, has a more restricted capacity to use exogenous cysteine, due to differences in metabolic pathways and transport (Bloom et al. 2022). Together these findings underscore the importance creating a better understanding of amino acid availability and usage in the vaginal environment.

### Intra-species variability within *Lactobacillus crispatus*

It is important to note that considerable intraspecies variation exists within *L. crispatus*, which may influence their nutrient requirements. Phylogenomic analysis, such as the study by Pan et al. (2020a), revealed distinct clustering among *L. crispatus* strains that correlates with their isolation source (e.g. poultry, human vagina and gut). This niche divergence was also reflected in different growth behaviour between strains (n = 4) from the human gut and vagina (Pan et al. 2020b). Even within strains isolated form the human vagina genetic and phenotypic differences have been previously observed, for example in their ability to degrade glycogen (Hertzberger et al. 2022). In the present study, only two vaginal strains were taken into consideration, whose phenotype may not fully represent the species as a whole; the results might reflect strain-specific characteristics. Consequently, caution is warranted when extrapolating these findings to other *L. crispatus* strains, especially when isolated from different environmental niches.

## Limitations of the study

While this study provides valuable data, some limitations should be considered. Cells were not washed between propagation steps, which may have allowed residual nutrients of the rich NYCIII pre-culture medium. It is thought that this bias is mitigated by performing at least two sequential propagations. Each step involved a 50-fold dilution, resulting in an effective NYCIII dilution of approximately 1:2500 in the second propagation and 1:125,000 in the third. The media design and experimental setup occasionally posed challenges in drawing definitive conclusions regarding the role of individual compounds. In some cases, conclusions were made cautiously or not at all. For example, while vitamin B12 was observed to be non-essential, this conclusion may not hold if methionine had been omitted, given the interdependence of their impact on metabolic routes (Hébert et al. 2004b). Therefore, the findings should be interpreted within the context of the specific media composition used in this study.

## Conclusion

A chemically defined medium for the growth of *L. crispatus* has been developed. On one hand, this body of work adds to the physiological understanding of lactobacilli and lays the groundwork for future quantitative studies on *L. crispatus*, on the other hand, we have shown that *L. crispatus* exhibits a high metabolic dependency on its environment, relying on exogenous fatty acids, essential B vitamins (e.g., riboflavin, folate), and all amino acids, highlighting its inability to synthesise these nutrients de novo and its potential sensitivity to nutrient variations and limitations impacting its ability to thrive in the vaginal microbiome.

## Supplementary Information

Below is the link to the electronic supplementary material.

Supplementary file1 (DOCX 4179 KB)

## Data Availability

Data is provided within the manuscript or supplementary information files.
