# Supplementary material for: Insights into host dependency from a chemically defined medium for the human vaginal bacterium Lactobacillus crispatus
Source: Arch Microbiol. 2025 Aug 6;207(9):212. doi: 10.1007/s00203-025-04406-z (PMC12328491; doi:10.1007/s00203-025-04406-z)
Supplement: Supplementary file 1 — Supplementary file1 (DOCX 4179 KB) [file 203_2025_4406_MOESM1_ESM.docx]

Supplementary material

This supplementary material is associated with the paper by Achterberg, et al., (2025). Insights into host dependency for a chemically defined medium for the human vaginal bacterium *Lactobacillus crispatus.*


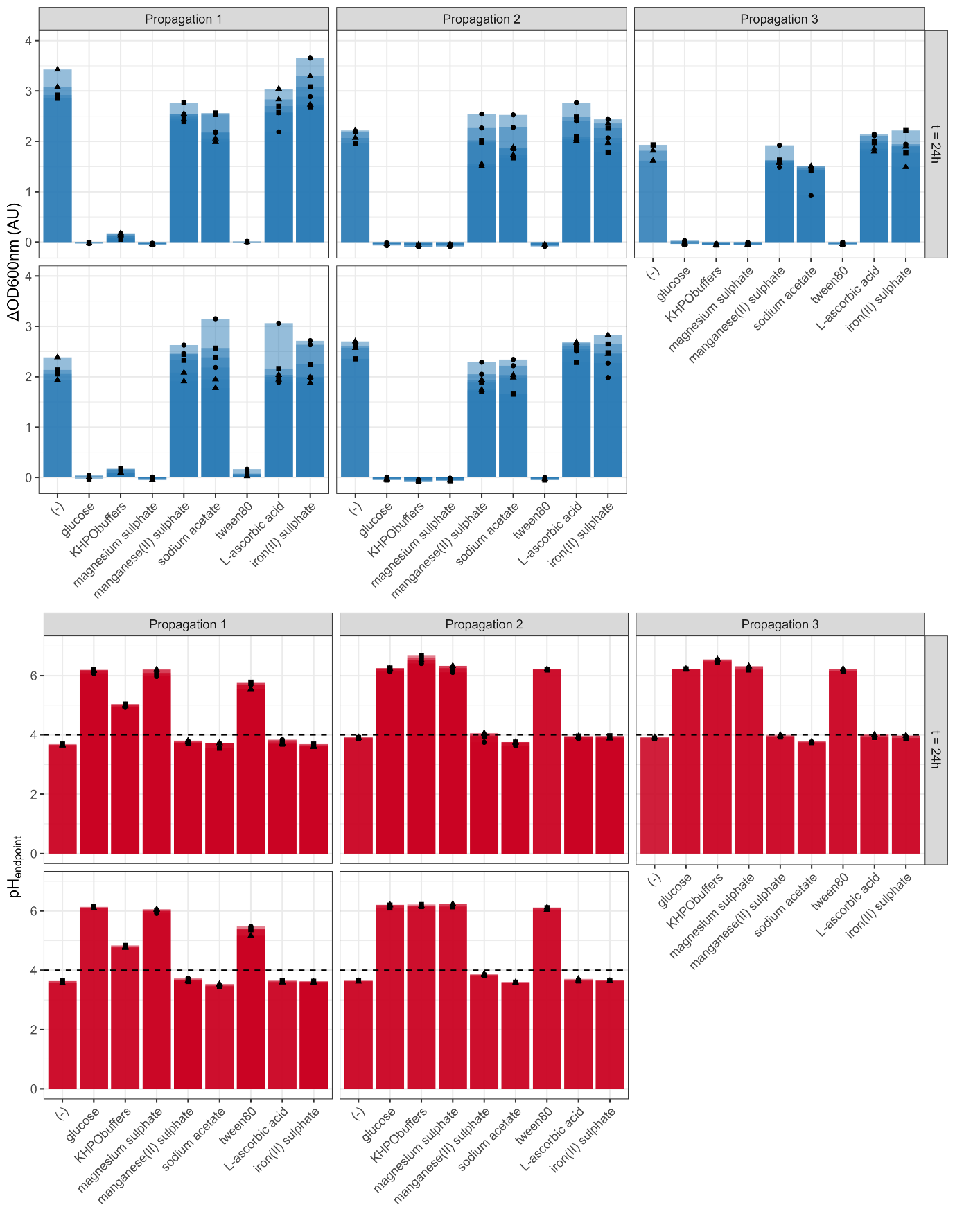


**Figure S1**. The effect of single and multiple omission of basal components of the chemically defined medium on the biomass growth and acidification of L. crispatus RL10. Three biological replications are shown (● , ▲ and ■), each comprising the average of two or three technical replicates. The X-axis depicts the omitted nutrient, with (-) being the complete CDM. Top: the Y-axis the increase in optical density at 600nm (OD600nm). Bottom: the Y-axis represents the measured pH at the end of the batch (pHendpoint).


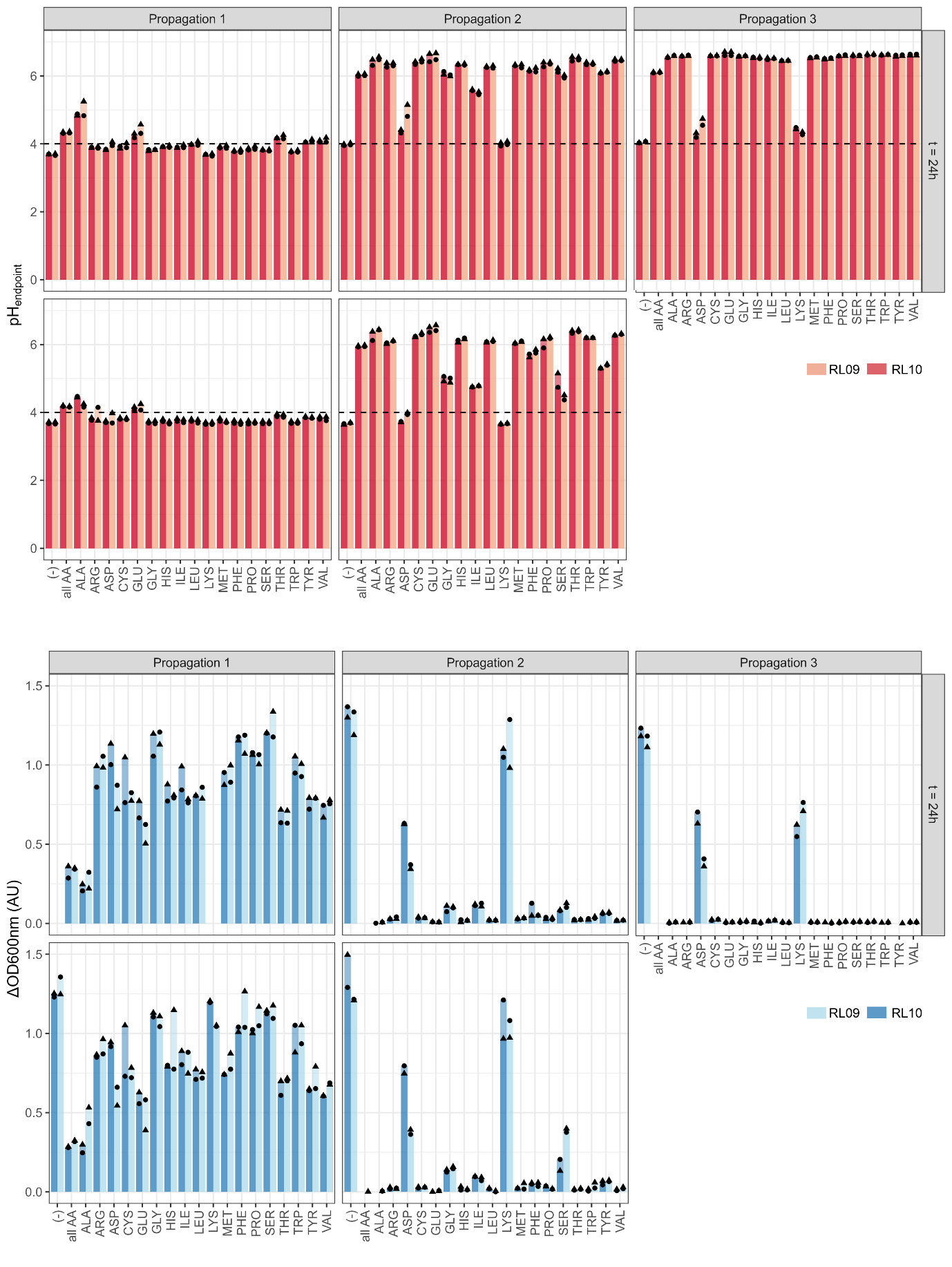


**Figure S2**. The effect of the single or complete omission of amino acids of the chemically defined medium on the biomass growth and acidification of L. crispatus RL09 and RL10. Two biological replications (● and▲) are shown. The X-axis depicts the omitted nutrient, with (-) being the complete CDM. Top: the Y-axis depicts the increase in optical density at 600nm (ΔOD600nm). Bottom: the Y-axis represents the measured pH at the end of the batch (pHendpoint).


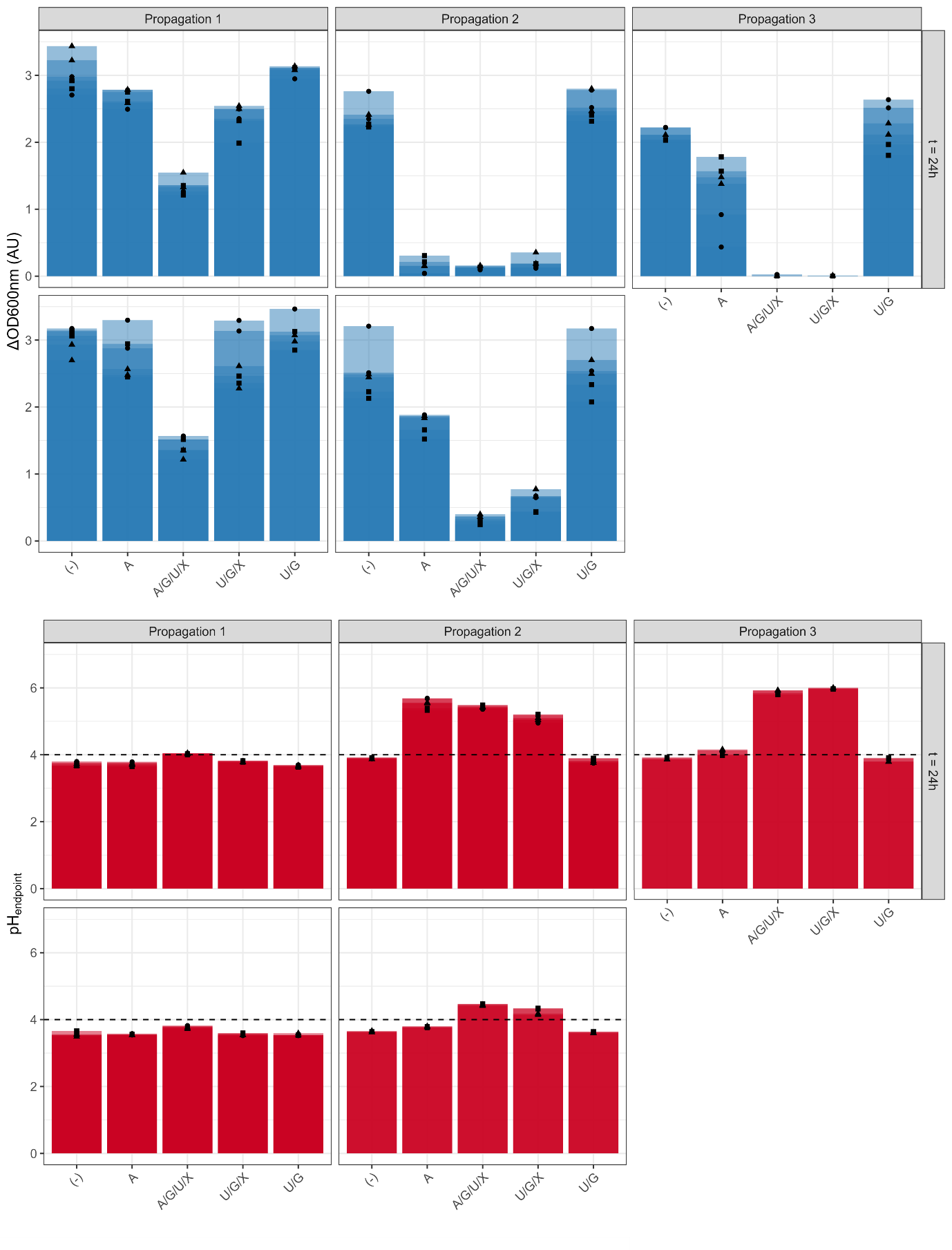


**Figure S3**. The effect of the single or complete omission of nucleotides of the chemically defined medium on the biomass growth and acidification of L. crispatus RL10. Two biological replications (●, ▲ and ■) are shown including their technical replicates. The X-axis depicts the omitted nutrient, with (-) being the complete CDM. A – adenine, G – guanine, U – uracil, X – xanthine. Top: the Y-axis depicts the increase in optical density at 600nm (ΔOD600nm). Bottom: the Y-axis represents the measured pH at the end of the batch (pHendpoint).

**
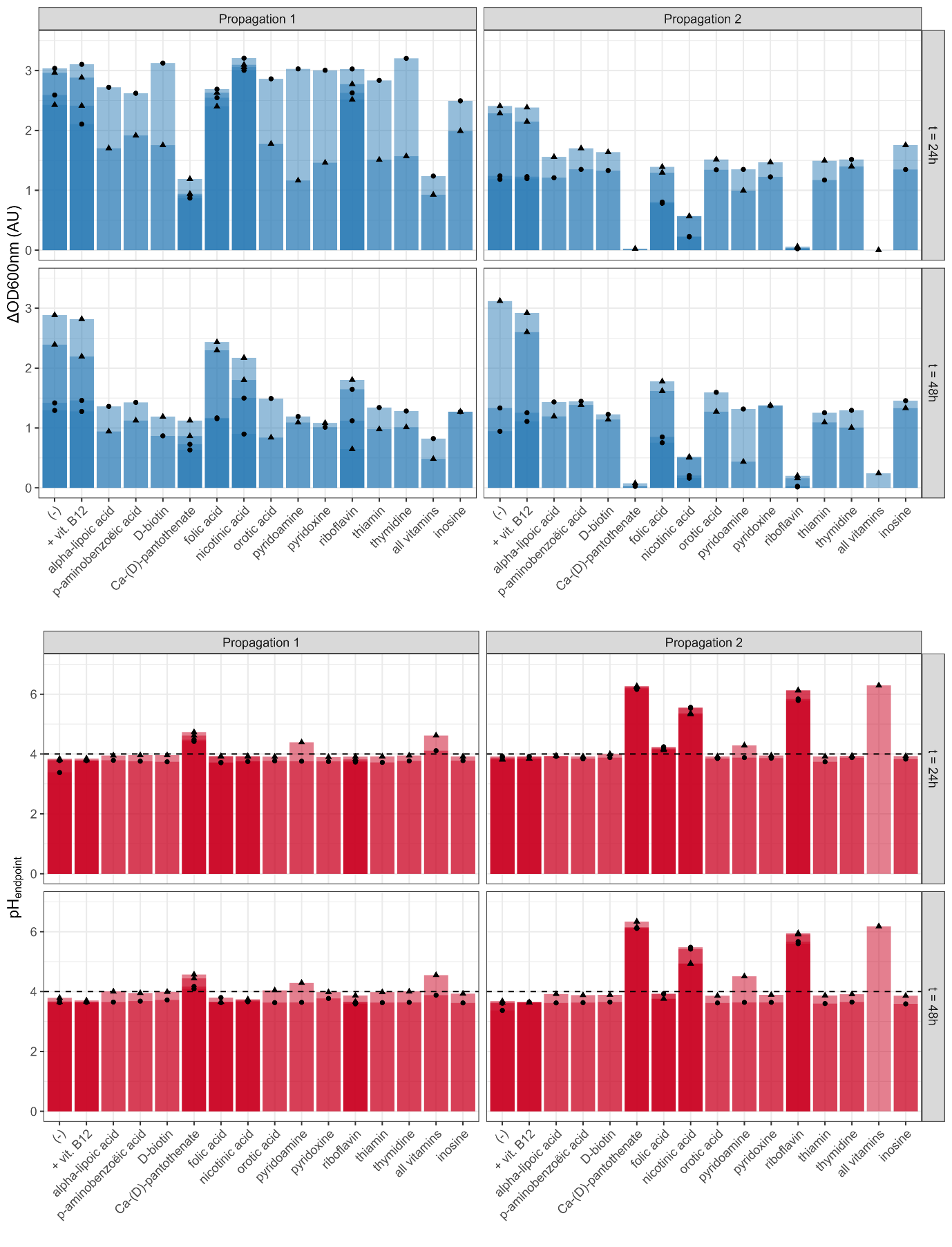
**

**Figure S4**. The effect of the single or complete omission or addition (vit. B12) of vitamins/nucleotides of the chemically defined medium on the biomass growth and acidification of L. crispatus RL10. Two biological replications (● and▲) are shown including their technical replicates. The X-axis depicts the omitted nutrient, with (-) being the complete CDM or addition to the cCDM for vit. B12. Top: the Y-axis depicts the increase in optical density at 600nm (ΔOD600nm). Bottom: the Y-axis represents the measured pH at the end of the batch (pHendpoint).
